# Learning from Acceleration Data to Differentiate the Posture, Dynamic and Static Work of the Back: An Experimental Setup

**DOI:** 10.3390/healthcare10050916

**Published:** 2022-05-15

**Authors:** Elena Camelia Muşat, Stelian Alexandru Borz

**Affiliations:** Department of Forest Engineering, Forest Management Planning and Terrestrial Measurements, Faculty of Silviculture and Forest Engineering, Transilvania University of Brasov, Şirul Beethoven 1, 500123 Brasov, Romania; elena.musat@unitbv.ro

**Keywords:** Industry 4.0, artificial intelligence, machine learning, job-related disorders, back, posture, dynamic, static, classification, performance

## Abstract

Information on body posture, postural change, and dynamic and static work is essential in understanding biomechanical exposure and has many applications in ergonomics and healthcare. This study aimed at evaluating the possibility of using triaxial acceleration data to classify postures and to differentiate between dynamic and static work of the back in an experimental setup, based on a machine learning (ML) approach. A movement protocol was designed to cover the essential degrees of freedom of the back, and a subject wearing a triaxial accelerometer implemented this protocol. Impulses and oscillations from the signals were removed by median filtering, then the filtered dataset was fed into two ML algorithms, namely a multilayer perceptron with back propagation (MLPBNN) and a random forest (RF), with the aim of inferring the most suitable algorithm and architecture for detecting dynamic and static work, as well as for correctly classifying the postures of the back. Then, training and testing subsets were delimitated and used to evaluate the learning and generalization ability of the ML algorithms for the same classification problems. The results indicate that ML has a lot of potential in differentiating between dynamic and static work, depending on the type of algorithm and its architecture, and the data quantity and quality. In particular, MLPBNN can be used to better differentiate between dynamic and static work when tuned properly. In addition, static work and the associated postures were better learned and generalized by the MLPBNN, a fact that could provide the basis for cheap real-world offline applications with the aim of getting time-scaled postural profiling data by accounting for the static postures. Although it wasn’t the case in this study, on bigger datasets, the use of MLPBPNN may come at the expense of high computational costs in the training phase. The study also discusses the factors that may improve the classification performance in the testing phase and sets new directions of research.

## 1. Introduction

Evaluating working postures [1] and particularly their dynamics in space and time [2] is essential in many disciplines, including for understanding the relation between human physical workload and the risks of developing work-related musculoskeletal disorders. An important body of knowledge has been devoted to evaluating the types of postures people assume during their work, mainly because some of them can lead to pain in various parts of the body [3]. While there is a high chance for a given individual to develop a work-related musculoskeletal disorder during his (her) life, poor body postures during work may often be constrained by the nature of the work itself, such as the characteristics of the tasks or poor ergonomic design of jobs and workstations [3]. In addition, other constraints may exist due to the interaction between the worker, the work object, and the environment as specific to some jobs [4]. Accordingly, the assessment of body postures has become increasingly important to the fields of ergonomics and human health, mainly because its results are helpful in the design and improvement of jobs and workplaces [3], but also because such results can be linked to those of epidemiological studies [5,6,7,8,9,10,11] with the aim of balancing biomechanical exposure and improving the general work environment. Understanding the effects of variability and diversity in biomechanical exposure is equally important to bring evidence on the best postural profiles for given jobs [2]. Such an attempt would probably need to integrate the results of epidemiological studies, and there is reason to think that it would require some means for handling accurately labeled long-term data.

Several methods have been developed, tested, and validated to evaluate the postural conditions of given jobs [12], and statistical improvements were added to some of them so as to enable comparability of their outputs in terms of postural diversity [13]. Although useful and widely used, many of them still share an important limitation, namely that of using samples that are often limited in size, mainly due to the effort required to collect, analyze, and interpret the data. On the other hand, small sample sizes often affect the precision of results, irrespective of the strategy used in data sampling [14,15].

The development in new sensing modalities and machine learning has enabled a diversification of the methods used in postural assessment. Computer vision has become one of the methods used for long-term data collection and to run analytics based on video footage and imagery, although the examined activities and postures have frequently been characterized by low complexity. Examples of studies and reviews on using computer vision for ergonomic-postural assessment include, for instance, those of [16,17,18,19]. Only a few of them actually addressed the whole body, or complex tasks, while the use of machine learning techniques for classification has often led to rather low classification accuracies. Chan et al., 2020, for instance, have found classification accuracies of 80 to 90% when extracting and using the key joints of the human body and data augmentation. Fernandez et al. [19] have checked the agreement of RULA (Rapid Upper Limb Assessment) scores provided by a computer vision and machine learning approach with expert-produced ones. Although they found a good agreement for some tasks, as measured by the Cohen’s k, in general this metric was around 0.6.

With the development of direct measurement methods, including those based on the use of accelerometers, new opportunities have emerged for collecting, processing, and analyzing data in the long term [20] under the umbrella of human activity recognition [21]. A variety of sensor systems have been tested for such purposes, including those used to gain knowledge on body posture and biomechanics [22]. In addition to using them to recognize and classify regular human tasks, several other useful applications were approached, including some deployed in particularly complex work environments characterized by human–tool interactions [23,24]. Acceleration signals, in particular, have been found to be very useful in mapping the intensity of human activity events in the time domain (e.g., [25]). Although the problems related to intra- and interclass variability and similarity still persist [21], such signals have the ability of creating patterns in magnitude that are useful in delimitating and statistically learning specific events, particularly when the data has been collected multi-modally. For instance, the work of [26] has shown the utility of multi-modally collected acceleration data in developing a system for correctly lifting weights, while the work of [27] describes a wide spectrum of techniques that were used to make accelerometer data suitable for human activity recognition. 

Similar to the computer vision approach to the problem, the performance of classification seems to be affected by the complexity of tasks and by the complexity of the classification experiment. Hu et al. [28] have used an electromagnetic motion-tracking system and a neural-network-based deep learning approach to recognize the prevalence of lower back pain. In their experiment, only static standing tasks were considered, reaching 97.2% in classification precision and recall. For a three-class problem and based on signals collected by accelerometers and gyroscopes integrated in smartphones, Nath et al. [29] have reached a classification accuracy of 90.2%, based on an implementation of a support vector machine (SVM) machine learning algorithm. Lifting tasks were also examined by means of inertial measurement units placed on various parts of the body and machine learning, yielding an accuracy of 99.4% for a two-class problem [30]. However, the classification accuracy was diluted significantly (76.9%) as the complexity of classification increased. All of these demonstrate the difficulty in correctly classifying complex body postures, which formed a centerpiece of evaluation in most studies and which may increase mainly as an effect of diversity in activities or tasks. On the other hand, the sequence of body postures in a given activity or task may be quite complex, a fact that may add to the dilution in the classification performance. With regard to the detection of static and dynamic work, based on acceleration data, Hosseinian et al. [31] have reached classification accuracies of 93–98.2% and 95.5% with random forest and support vector machine algorithms, respectively. However, their work was focused mainly on activities and not on body postures.

The possibility of using acceleration signals to classify the main postures of the back, while differentiating between static and dynamic work by statistical learning, would bring many benefits to the science and practice of several fields. On the one hand, such a system could be deployed to evaluate the postures assumed by given subjects in the time domain, which would be useful in collecting and analyzing long-term postural data to support ergonomic interventions and healthcare. Meanwhile, if such a system could separate the dynamic from static work in the time domain, it would be useful in profiling the variability and diversity of postural change as specific to given jobs. We acknowledge here the previous work done to test the effectiveness of activity recognition by machine learning techniques applied to acceleration signals. Such work has focused, for instance, on recognizing dynamic and static regular tasks [32] or on activity recognition in supervised and unsupervised trials [33]. The suitability of using acceleration signals and machine learning techniques to correctly detect postures in complex sequences of movements or activities while accounting for the type of work has been less studied, making the latest approach to activity recognition less suitable to postural analysis since many of the tasks are built upon a sequence of changing postures. In addition, the same work tasks may be developed at different paces by the same or different individuals (e.g., [25]), which may result in a high variability of body postures and dynamic and static work. The upper part of the body, and in particular the back, has been documented to be of a serious concern in terms of the association between working postures and musculoskeletal disorders (e.g., [34,35,36,37,38]). Accordingly, this would require a closer eye on the variability in working postures and dynamic and static work of the back, which means that high amounts of data would be needed to extract causal information to explain various kinds of disorders. As the collection of this data has been enabled by the availability of low-cost sensors (e.g., [23,24,25]), the only problems that still need to be overcome in extracting useful information from the data are those related to labelling and finding reliable algorithms to learn and generalize from them.

The goal of this study was to test whether it is possible to differentiate between static and dynamic work while correctly identifying the postures of the back assumed by a subject, by a machine learning approach to the data collected by a triaxial accelerometer in a controlled setup. Accordingly, the objectives of this study were: (i) to identify and apply a suitable denoising procedure to the accelerometer data; (ii) to identify the most suitable machine learning algorithms to classify the data on back posture and dynamic and static work from a set of two choices, namely neural networks and random forests; and (iii) to use the most high-performing machine learning algorithms to learn and generalize on the data of postural, dynamic, and static work of the back.

## 2. Materials and Methods

### 2.1. Experimental Design and Data Collection

**Figure 1:**
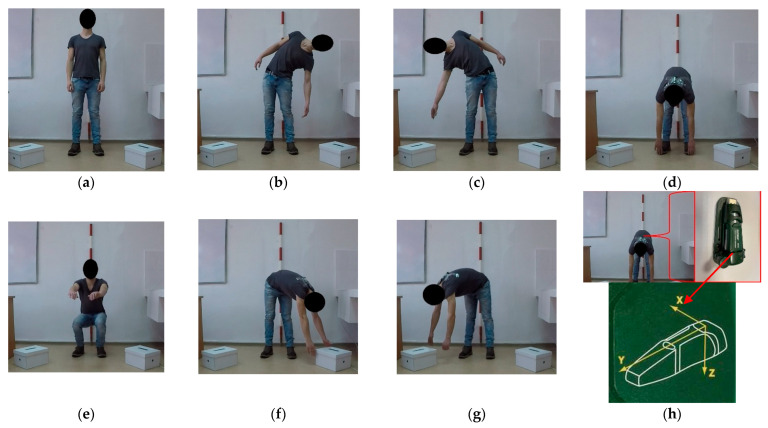
Description of the main postures of the back reached and maintained by the subject during the implementation of the movement protocol: (**a**) orthostatic posture maintained before each movement subprotocol and between the repetitions of a given subprotocol; (**b**) back bent to the left of the subject; (**c**) back bent to the right of the subject; (**d**) back bent forward; (**e**) knees bent, back straight; (**f**) back bent forward and twisted to the left; (**g**) back bent forward and twisted to the right; (**h**) placement of the datalogger, and the datalogger used to collect acceleration data, including axis orientation.

An experimental protocol was developed in this study, with the main aim of collecting data for the static and dynamic work, by taking into consideration the main postures that one may assume for the back. A voluntary healthy subject (25 years old, 68 kg in weight, and 179 cm in height) agreed to mimic the back movements as described by the protocol (Table A1), based on informed consent and free will to participate in the study. The subject was instructed to make his movements at full extent and at low speed with the main aim of facilitating data labelling. Each type of movement was preceded by maintaining an orthostatic posture (Figure 1a) for a certain amount of time (Table A1), followed by bending the back (to the left and right side of the body at full extent, Figure 1b,c), maintaining the bent posture of the back for a given amount of time (Table A1), and returning to orthostatic posture, which was also maintained for a given amount of time (Table A1). Similar procedures were followed by the subject to bend his back forward, bend his knees (back straight), and to bend and twist his back forward to the right and left, respectively (Figure 1d–g), resulting in six subprotocols of movement. By excluding the orthostatic posture, a given movement and posture maintained was repeated ten times in a continuous sequence for each movement type, resulting in a total of 60 repetitions (10 repetitions for each subprotocol). 

A painted rod was used to point the vertical, to align the subject to the orthostatic posture and to guide a researcher that observed the movements done by the subject (Figure 1). Two paper boxes were used to help the subject self-guide in his movements of bending and twisting the back forward and sideways (Figure 1f,g). The boxes were placed on the floor at angles of ca. 45° from the plane of the room wall, which was taken as reference for the experiment. During the implementation of the movement protocol, the subject was guided by the researcher regarding the beginning and end of each movement, as well as on the time needed to maintain a given posture, which was done with the help of the time counter of the video camera used to record the experiment. The camera used was a GoPro Hero 10, which was placed on a table at ca. 3 m from the subject and set to continuously record all the events of the experiment in a narrow field of view, at 60 frames per second and at a resolution of 1080 pixels.

Acceleration data was collected by an Extech^®^ VB300 (Extech Instruments, FLIR Commercial Systems Inc., Nashua, USA v2017, Figure 1h) triaxial acceleration datalogger, which was placed on the back of the subject in between the scapulae, close to the junction of the thoracic and cervical vertebrae. The datalogger was attached to the subject’s shirt by adhesive tape, and it was placed so that the y-axis was oriented towards the vertical plane, from the bottom to the top of the room; the x-axis was on the horizontal, in the right-to-left plane, parallel to the room’s wall; and the z-axis was on the horizontal, in the front-to-back plane, which was perpendicular to the room’s wall. Before placing it on the subject, the datalogger was set up with dedicated software to continuously collect data at a rate of 20 Hz, in normal record mode and by manual start. Once the video camera was started, the datalogger was placed on the subject, it was started, and the experiment was implemented according to the described protocol.

### 2.2. Data Processing

Following the experiment, the video footage and data collected by the accelerometer were downloaded and stored in a personal computer. Video footage was available in the MP4 format, while the accelerometer data was exported in a tabulated form supported by the Microsoft Excel^®^ (Office 2021 Professional, Microsoft, Redmond, WA, USA) .xls format, along with an ID and a time label.

**Figure 2:**
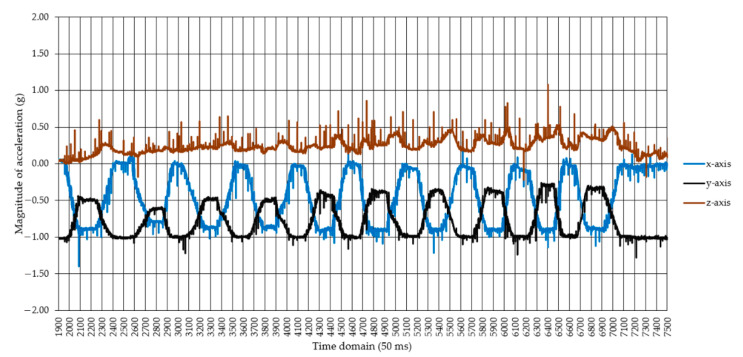
An example of the raw data collected by the triaxial accelerometer for the subprotocol A1, involving back bending to the left of the subject: the x, y, and z axes in the legend stand for the responses (g) on x, y, and z axes, respectively; right end part of the figure (from 7050 to 7500 50-ms increments) shows in the responses on the three axes during part of the time in which the orthostatic posture was maintained; relatively static responses in the figure indicate the time in which a given posture was maintained; increments and decrements in magnitude indicate the time in which movements were done to change a given posture. Note: the signal contained impulse noise and oscillations.

In total, 34,639 observations were retained for processing from the data collected during the experiment, in the form of the magnitude of responses of the x, y, and z axes (Figure 2), including the Euclidian norm and a date and time label for each observation sampled at 20 Hz. Video data, as well as the magnitude of acceleration and changes in it, were used as a reference to label each observation according to the real events that occurred during the experiment, and codes were given to each of the observations recorded as triaxial acceleration responses. For this purpose, a coding procedure was developed (Table 1) to account for the posture of the back and legs and the presence or absence of movement (dynamic vs. static work). The tasks described in Figure 1 and Table 1 and Table A1 were chosen with the aim of covering the most common degrees of freedom characterizing the potential movements of the back, therefore on generalization criteria. The concept used to differentiate between dynamic and static work was as follows: each instance in which any kind of intended movement was done according to the described protocols was classified as dynamic work, while keeping a given posture, including the orthostatic one, was classified as static work.

For example, if a given observation was found to belong to the subject’s movement to bend his back to the left, then the attributed code was BblLsPt (B—back, bl—bent to the left, L—legs, s—straight, P—presence of movement, t—true); orthostatic postures were characterized by the code BsLsPf. Prior to labelling, based on the media files and the patterns in the magnitude of acceleration data, the periods of placing and taking down the accelerometer from the subject were removed, and only the data covering the protocols were retained.

### 2.3. Noise Removal

Providing an enhanced capability for a machine learning algorithm to accurately classify based on an acceleration input signal may require processing procedures to minimize intra-class variability and inter-class similarity. Acceleration signals integrate gravitational, movement, and noise components (e.g., [39,40]). In static conditions, the vector magnitude (Euclidian norm) of triaxial acceleration is, ideally, close to 1 [41]. However, movement adds to the responses in magnitude [40] on the relevant axes, while temperature may offset the readings, depending on the study location [41]. Among the techniques used to remove the noise from the acceleration signals, median filtering has been proven to be very useful, mainly because it helps preserve the location of inter-class edges in the time domain. A basic description of the median filter and its properties in altering the geometry of a signal is given, for instance, in [42]; accordingly, median filters hold the capability of removing impulse noise and reducing oscillations at the first pass of the signal through the filter. The extent to which noise can be removed depends largely on the quality and characteristics of the signal, as well as on the size (sliding window) of the median filter. This study used an iterative, trial-and-error procedure, by applying median filters of various odd sizes over the triaxial data, starting from a window size of 3. Following each iteration, the filtered data were plotted and visually compared against the original data collected on the three axes. The lowest widow size at which the filter succeeded in removing impulses and oscillations was kept as final, and the resulting data were prepared for testing the performance of machine learning algorithms.

### 2.4. Selection of Machine Learning Algorithms

Two machine learning algorithms were tested to check the learning performance over the filtered datasets, namely a neural network and a random forest. Both machine learning algorithms were tested in Orange Visual Programming software [43], version 3.31.1, which enables the use of several machine learning classifiers under a widget-based, visual architecture.

A multi-layer perceptron with backpropagation neural network (MLPBNN) algorithm was used as a first learning option. Following the results and recommendations found in relevant literature [44,45], the number of hidden layers and of neurons per hidden layer were set at the maximum enabled by the used software (3 and 100, respectively), in an architecture of fully connected layers. In addition to the width and depth, MLPBNN algorithms require tuning for several parameters, such as the activation function, solver, regularization parameter (α, L2 penalty norm), and number of iterations. The software also provides functionalities to train and test the algorithm by cross-validation. Based on previous experience with the acceleration data collected by the same type of datalogger [24,46], as well as on findings related to the performance of activation functions such as the rectified linear unit (ReLU) and solvers such as the stochastic gradient-descent-based optimizer (ADAM) [44,45,47,48,49], these two hyperparameters were kept the same during the tests. Number of cross-validation folds was set at 5, and the maximum number of iterations at 1,000,000. The regularization term (α) was set successively at 0.0001, 0.001, 0.01, 0.1, 1, 10, and 100. The actual tests were done over all the filtered data (34,639 observations collected at 20 Hz) by an axis-based fusion [50], which involved feeding the filtered signals of each axis as input features to the MLPBNN. The number of tests carried out was 14. This accounted for seven values set successively for the regularization term, and for the two problems pursued by the study, namely: i) to evaluate the performance of classifying the dynamic and static work (2 classes); and ii) to evaluate the performance of classifying the posture of the back (13 classes), which accounted also for the presence or absence of movement.

Random forest (RF) is an ensemble learning algorithm that was first proposed by Ho [51] as an effective algorithm for problems involving high dimensionality of data. It was then further developed by Breiman [52] and is currently used for classification and regression problems. Random forests build on a combination of tree predictors in such a way that each tree depends on the values of a random vector sampled independently, and the generalization error converges when the number of trees is very large [52]. More specifically, RF builds a set of decision trees, where each tree is developed from a bootstrap sample collected from the training data; for each tree, a subset of attributes is drawn to evaluate which one is the best for decision making, and the final models builds on a majority voting coming from the trees. RF has the advantage of using fewer hyperparameters, working well on highly dimensional data, and training quickly. In Orange Visual Programming software, two categories of parameters can be tuned, namely the basic properties, among which the number of trees and attributes used at each split are important, as well as the tree growth parameters. The same number of tests was carried out using RF, where the number of attributes used at each split was kept at the default setting provided by the software, while the smallest subset for splitting was set at 5. Number of cross-validation folds was kept the same, as in the case of the MLPBNN algorithm. The only tuned parameter was the number of trees, which was set successively at values of 10, 50, 100, 500, 1000, 5000, and 10,000.

Selection of the best performing ML algorithms was based on the performance metrics described in Section 2.6. From each class of ML algorithms (MLPBNN, RF), the best performing ML architectures were selected based on the highest classification accuracy and recall and on the lowest cross-entropy, respectively. The final best performing architectures were compared against each other based on the same performance metrics and the best-performing ones were kept as final for training and testing purposes.

### 2.5. Training and Testing

**Figure 3:**
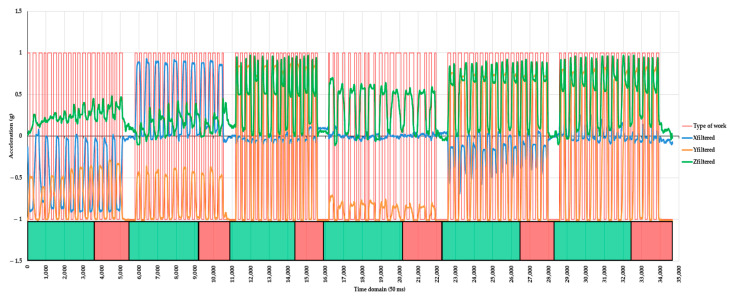
Data partitioning into training (green strips at the bottom of the figure) and testing (red strips at the bottom of the figure) data subsets. Legend: Xfiltered, Yfiltered, and Zfiltered are the values of the signals on the three axes after applying a median filter with a window size of 41; type of work is an arbitrary signal taking a value of 1 when dynamic work (movement) was present and −1 when static work (no movement) was present.

For training and testing purposes, the data was split into two subsets, of which the training one was designed to include the data of the first seven movements of each subprotocol (Table A1), including half of the data characterizing the time spent in orthostatic posture between each subprotocol. The rest of the data, including the last segment of the orthostatic posture following all the subprotocols, were attributed to the testing subset. Figure 3 shows the concept used to divide the data into training and testing subsets.

Following the extraction from the filtered dataset, data corresponding to each subset were merged into a specific file, resulting in two files, one of which was used for training and the other for testing purposes. Training and testing phases were done in Orange Visual Programming software by using the widgets and following the steps described in Section 2.6. Original data, filtered data used to select the best-performing algorithms, and data divided into training and testing subsets were stored in Microsoft Excel^®^ spreadsheets. Details on data processing, filtering, summarization, and artwork development in Microsoft Excel^®^ are given in Section 2.6.

### 2.6. Performance Metrics Used in Evaluation, Software Used, and Computer Architecture

There are many metrics that could be used to evaluate the performance of classification. A detailed description of those relevant in classification applications by machine learning can be found, for instance, in [53,54]. In this study, the classification accuracy (hereafter CA), recall (hereafter REC), and cross-entropy (hereafter LOGLOSS) were used as metrics to (i) choose the best models for training and testing and (ii) evaluate the classification performance in the training and testing phases. Classification accuracy stands for the ratio of true positive and true negative instances that have been correctly classified to the total number of instances (true positive, true negative, false positive, and false negative) [54], recall is the ratio of true positives classified as such to the true positives and false negatives [53,54], and cross-entropy (LOGLOSS) is the negative log-likelihood of a logistic model that returns the probability of an instance being correctly classified [55].

All the steps used to process the data were done on a personal computer equipped with the following software and hardware: system type—Alienware 17 R3 (Dell Inc., Round Rock, TX, USA); processor—Intel^®^ Core™ i7-6700HQ CPU, 2.60GHz, 2592 MHz, 4 cores, 8 logical processors; installed physical memory (RAM)—16 GB; operating system—Microsoft Windows 10 Home. Data exported from the dedicated software of the triaxial accelerometer were stored in a Microsoft Excel^®^ spreadsheet. The same software was used to label the data, to apply and evaluate the effects of median filters of different sizes, to store and analyze the performance metrics produced by Orange Visual Programming software [43], and to build most of the graphics used in this study. Orange Visual Programming software was used in all the steps that required work with ML algorithms. To support the step of selecting the best ML algorithms, the required processing workflows were built by interconnecting the Data, Neural Network, Random Forest, and Test & Score widgets. The same approach was taken to train the data for the architectures of the best ML models, supplemented by the Save Model widget to store the trained models. The testing phase involved the application of the saved models to the testing data subset using the Data, Load Model, and Predictions widgets, in conjunction with the Confusion Matrix widget, and data were exported to Microsoft Excel^®^, where more detailed analyses were done to identify and explain misclassifications.

## 3. Results

### 3.1. Characterization of the Input Dataset and Signal Filtering

The input dataset contained 34,639 observations, of which 16,224 (ca. 46.8%) characterized dynamic work, and the rest (18,415, ca. 53.2%) static work. The slight imbalance between these two classes was due to those instances characterizing the time spent in orthostatic posture between the subprotocols (as described in Table A1) and at the end of the experiment, as shown, for instance in Figure 3.

**Figure 4:**
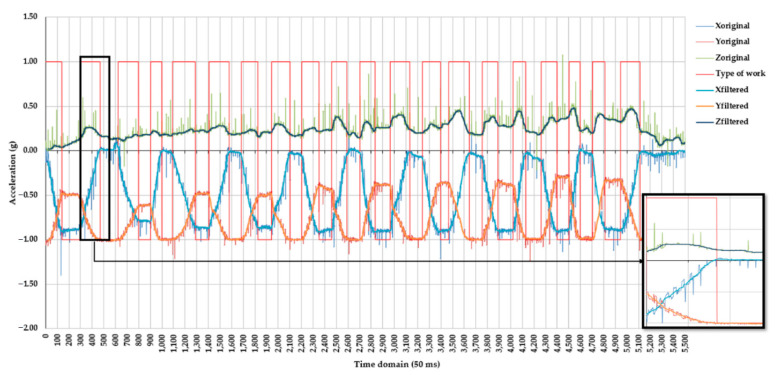
Effect of axial signal filtering by a median filter with a window size of 41. Legend: Xoriginal, Yoriginal, and Zoriginal are the original values collected by the accelerometer on the x, y, and z axes, respectively; Xfiltered, Yfiltered, and Zfiltered are the values of the signals on the three axes after applying a median filter with a window size of 41; type of work is an arbitrary signal taking a value of 1 when dynamic work (movement) was present and −1 when static work (no movement) was present. Note: the time domain from 0 to ca. 5100 (50 ms increments) corresponds to the subprotocol A1 (Table A1), and the time domain from ca. 5100 to 5500 (50 ms increments) (right part of the figure) corresponds to keeping an orthostatic posture before starting subprotocol A2; details given in black box indicate the utility of the used median filter in removing both impulse noise and oscillations during dynamic work.

Several window sizes of the median filter were tested over the original signals collected on the three axes by the accelerometer. Using a sliding window size of 41 for median filtering seemed to be the best strategy to remove the impulse noise on all the three axes, as shown in Figure 4, which compares the original values recorded on the three axes to their filtered counterparts. From the experience of applying several sizes of median filters on the original signals, it was observed that for static postures, median filters of smaller window sizes have successfully removed most of the impulse noise. However, for dynamic parts of the signal, lower window sizes failed to remove the oscillations caused by the movements done by the subject; by using a filter size of 41, these oscillations were removed from those signal parts characterizing the dynamic work, as shown in Figure 4 (black box on the right side). By removing the impulse noise and oscillations, which characterize largely intra-class variability, the used window size of the median filter was successful in providing an altered signal so as to enhance inter-class separability.

### 3.2. Selection of the Machine Learning Algorithms

**Figure 5:**
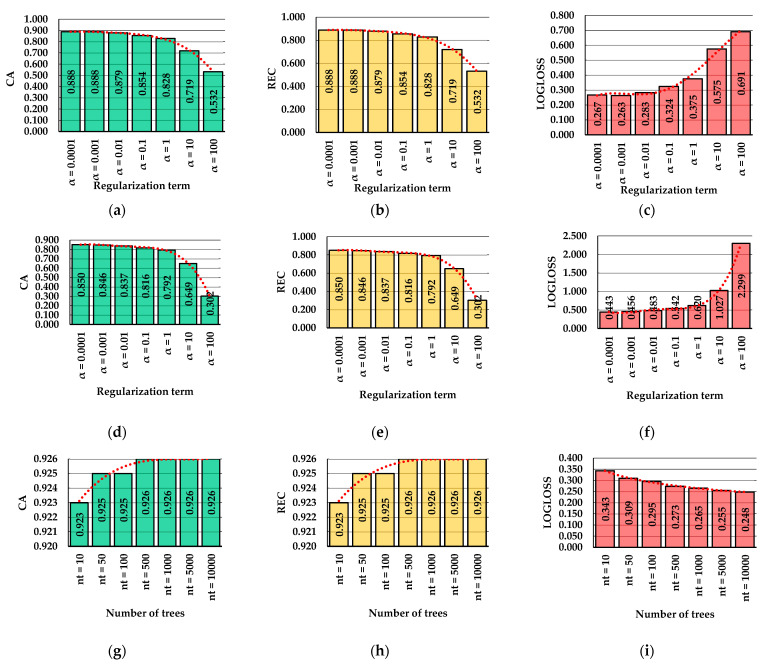

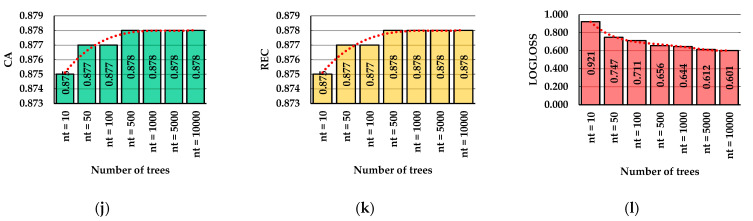
Variation in classification accuracy (CA), recall (REC), and cross-entropy (LOGLOSS) by parameter tuning: (**a**–**c**) results of the two-class problem of evaluating the classification performance over dynamic and static work by the MLPBNN as a function of the regularization term; (**d**–**f**) results of the 13-class problem of evaluating the classification performance over back postures by the MLPBNN as a function of the regularization term; (**g**–**i**) results of the two-class problem of evaluating the classification performance over dynamic and static work by the RF as a function of the number of trees; (**j**–**l**) results of the 13-class problem of evaluating the classification performance over back postures by the RF as a function of the number of trees.

Figure 5 summarizes the main results regarding the variation of the selected classification performance metrics (CA—classification accuracy; REC—recall; and LOGLOSS—cross-entropy) as a function of values selected for hyperparameter tuning. Figure 5a–c shows the results of classification accuracy, recall, and cross-entropy for the MLPBNN model trained to check the classification performance of separating the data into two classes, namely dynamic and static work of the back. For fine-tuned regularization parameters (α = 0.0001 and 0.001, respectively), the achieved classification accuracy (CA, standing for correctly classified instances) was high, accounting for ca. 89%. For the same conditions, the recall (REC) reached similar values, and the training error (LOGLOSS) accounted for 26.7% and 26.3%, respectively. Accordingly, as a first observation, the best outcomes in terms of classification performance of the MLPBNN machine learning algorithm when trying to differentiate between dynamic and static work of the back were achieved for a regularization term α set at 0.001 (CA = 88.85; REC = 88.85; LOGLOSS = 26.35).

In Figure 5d–f, the classification performance results are shown for the MLPBNN model trained to differentiate between the 13 classes characterizing the postures of the back, by accounting for the type of work (dynamic or static). Although the results of the classification accuracy (CA, ca. 85%) and recall (REC, ca. 85%) were promising for α set at 0.0001 and 0.001, respectively, the values of cross-entropy (LOGLOSS) increased significantly, being almost doubled in comparison to those of the model trained to differentiate between the dynamic and static work of the back. Beyond α set at 0.001, both CA and REC presented lower values, and LOGLOSS increased substantially. Up to α = 1, the area under the receiver operating characteristics (ROC) curve (AUC, data not explicitly given herein) kept a relatively constant trend in values (AUC > 0.9) for both MLPBNN models, indicating that the classifier used was performing rather well for these settings of the regularization term. Beyond this value, the AUC values decreased accordingly, indicating a lower performance of the MLPBNN models. Training the first set of seven models (dynamic vs. static work, data not explicitly given herein) took around 5 h, while training the second set of seven models (13 back posture classes, data not explicitly given herein) took close to 6.5 h by the computer architecture described in Section 2.6. However, for both models, there were no trends in time spent as a function of the value of the regularization term.

The maximum classification accuracy (CA) for the two-class (dynamic vs. static work) RF model was achieved with 500 trees and preserved its value irrespective of further increases in the number of trees in the model (Figure 5g). In fact, in this case, CA was higher by almost 4% compared to its best counterpart from the MLPBNN model. Recall values (REC, Figure 5h) followed a similar trend as a function of the number of trees used to train the model, with the maximum value (92.6%) reached at 500 trees and preserved beyond this point. For the same RF model (Figure 5g–i), the cross-entropy (LOGLOSS) had the highest value when the number of trees was set at 10, and it decreased significantly to 24.8% when the number of trees was set at 10,000. Accordingly, the minimum LOGLOSS of the RF model was 1.5% less compared to its minimum counterpart from the MLPBPNN model.

For the 13-class problem (Figure 5j–l), the RF was consistent in providing a better classification performance (CA, REC, LOGLOSS) compared to the MLPBNN model developed for the same problem. Values of CA and REC were found to reach their maximum when the number of trees was set at 500 (87.8%); maximum values were preserved beyond this point, and the minimum LOGLOSS value was found when the number of trees was set at 10,000. Nevertheless, the value of LOGLOSS was still high, and it most likely indicated a higher classification error. The area under the ROC curve (AUC, data not explicitly given herein) was more consistent in values in the case of the RF models, irrespective of the number of trees. It took the minimum values when the number of trees was set at 10, a condition for which it accounted for 0.968 in the case of the two-class problem and for 0.979 in the case of the 13-class problem. Training time (data not explicitly given herein) varied proportionally with the number of trees used in the model, accounting for ca. 4 h for the first seven models and for ca. 3.5 h for the last seven models.

Since the MLPBNN models with the regularization parameter set at 0.001 and 0.0001 provided the best results for the 2- and 13-class classification problems, these models were selected for training and testing purposes from the MLPBNN class. Based on the results shown in Figure 5, the RF model class consistently provided a better classification performance irrespective of the classification problem. From this class of models, an RF machine learning algorithm with the number of trees set at 10,000 was further selected for the training and testing phases for both classification problems.

### 3.3. Classification Performance in the Training and Testing Phases

Table 2 gives an overview on the data included in the training and testing subsets, with a focus on the classes of dynamic and static work of the back. The filtered triaxial set contained 34,639 instances, of which, following the procedures described in Section 2.5, ca. 68% (23,625 instances) were used for training the machine learning models. The rest of the data (11,014 instances, ca. 32%) were used for testing purposes. 

The instances characterizing the dynamic and static work of the back were relatively well balanced in both subsets (Table 2), accounting for ca. 49% and 51% (training subset), and for ca. 42% and 58% (testing subset), respectively. The higher share of instances characterizing the static work of the back in the testing subset was due to the inclusion of the last part of the filtered dataset, which characterized the orthostatic posture at the end of the experiment (Figure 3). Altogether, there was a relative balance between the instances characterizing the dynamic (ca. 47%) and static (ca. 53%) work of the back in the initial dataset.

**Figure 6:**
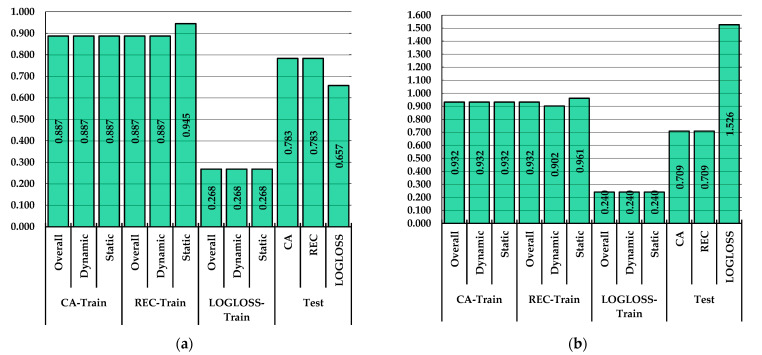
Classification performance in the training and testing phases to differentiate dynamic from static work: (**a**) by a MLPBNN machine learning algorithm with α set at 0.001; (**b**) by a RF machine learning algorithm with number of trees set at 10,000.

Figure 6 shows the results on the classification performance of the dynamic and static work of the back in the training and testing phases by the best performing model architectures of MLPBNN and RF as identified in Section 3.2. By a MLPBNN with α set at 0.001, the overall classification accuracy (CA, 88.7%) and recall (REC, 88.7) in the training phase were close in value to those from the pre-evaluation phase. At the class level, the REC metric returned a significantly higher value (94.5%) in the case of the static work. Cross-entropy error (LOGLOSS) was estimated at the same value irrespective of the class.

In the testing data subset, however, the classification performance decreased considerably, with CA and REC accounting for 78.3%, representing a dilution in classification performance of ca. 10%. Therefore, for the MLPBNN, this means that the algorithm performed better in the learning phase, but its ability to generalize was lower. Still, close to 80% of the data were correctly classified in the testing phase, which is a promising result given the complexity of the input signal. Similar trends in the classification performance were found for the RF model (Figure 6b), which, on the one hand, performed even better in the training phase, where it accounted for values of CA and REC of 93.2%. Taken on classes, the values of CA for the dynamic and static work were the same (93.2%), and similarly to the MLPBNN model, the REC metric of the static class was higher (96.1%). Cross-entropy error (LOGLOSS, 24%) also decreased in comparison to the pre-evaluation phase, and it had the same value irrespective of the class. In the testing phase, on the other hand, the results indicated a higher dilution of performance, with values of CA and REC of ca. 71%, which were lower by ca. 22% compared to the training phase. Based on the above, the MLPBNN algorithm performed slightly better in generalizing on the test data subset, although it returned lower values of classification performance metrics in the training phase. As such, it was more stable across the training and testing phases compared to RF.

**Figure 7:**
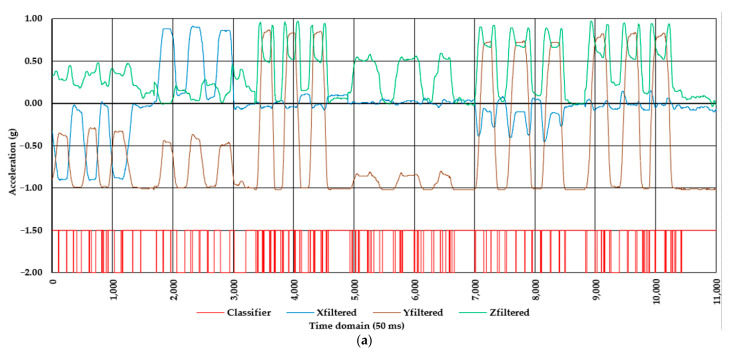

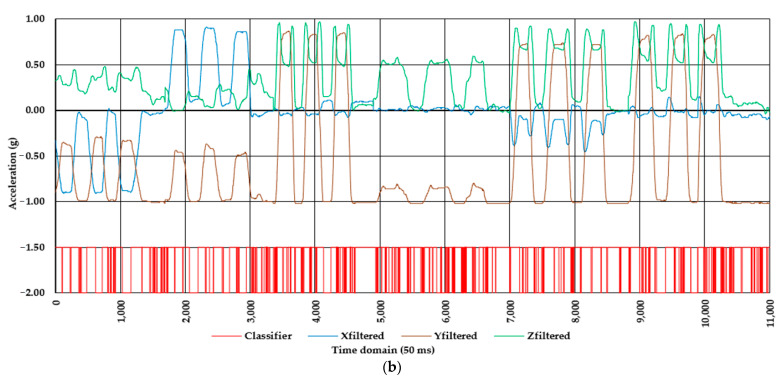
Misclassifications in the testing phase of the two-class problem (dynamic vs. static work of the back) by the two machine learning algorithms: (**a**) MLPBPNN (α = 0.001) algorithm; (**b**) RF (number of trees = 10,000) algorithm. Legend: classifier—an arbitrary signal showing the correct (acceleration = −1.5 g) and incorrect (acceleration = −2.0 g) classifications; Xfiltered, Yfiltered, and Zfiltered are the values of the signals on the three axes after applying a median filter with a window size of 41.

Figure 7 shows the patterns in data from the testing subset, along with the misclassifications found by comparing the actual and predicted classes of the MLPBNN (α = 0.001) and RF (number of trees = 10,000) algorithms when differentiating between the dynamic and static work of the back. For the MLPBNN model, which was taken as an example to explain the patterns in the data and their effects on the classification performance, the correctly classified instances accounted for 8,624, standing for ca. 78% of the testing data (Figure 7a, classifier = −1.5). In particular, the static work of the back was evenly correctly classified compared to the dynamic work. Correct classifications of the static work can be observed, for instance, at the right part of the figure where the orthostatic posture was kept at the end of the experiment, as well as in other parts of the figure where the values of the filtered signal of the y axis (Yfiltered) approached the value of −1, which meant that the body was kept in an orthostatic posture. 

In those cases where the data pattern showed constancy along the axis of the time domain, the events were correctly classified, with most of such instances representing static work. However, in some transition parts along the signals’ patterns, as well as in some points from the time domain in which such transitions began or ended (inter-class edges), the data have been misclassified. An example of intra-class similarity can be seen, for instance, in the time data range from ca. 3300 to ca. 4600 (50 ms increments) (Figure 7a). This data segment corresponded to the subprotocol A3 (bending back forward), which explains the data pattern in the Zfiltered signal, which was in contrast to most of the rest of the subprotocols. Accordingly, this time frame was the one characterized by the highest frequency of misclassifications (classifier = −2 g, Figure 7a), emphasizing the effect that intra-class similarity may have on the classification performance. Based on the data transferred to and interpreted in a Confusion Matrix widget, 932 (20% of the actual class) of the instances labeled as dynamic and 1458 (22% of the actual class) of those labeled as static work were misclassified.

Figure 7b, on the other hand, shows the pattern in misclassification in the case of the RF algorithm used to differentiate between the dynamic and static work. Although the patterns in misclassification were similar to those of the MLPBNN algorithm, the difference was in the frequency of misclassifications, which was obviously higher. The correctly classified instances accounted for 7826, representing ca. 71% of the testing data. Misclassifications of dynamic work decreased to approximately half (ca. 11% of the actual data on dynamic work) compared to those specific to the MLPBPNN algorithm. However, the difference in misclassification occurred mostly at the expense of classifying static as dynamic work (2715 instances, ca. 42% of the actual data on static work). An example of such misclassifications may be seen at the right part of Figure 7b (between 10,300 and 11,000), where the actual class was that corresponding to keeping an orthostatic posture of the body.

Results showing the training and testing performance of the two machine learning algorithms (MLPBNN and RF) on the 13-class postural problem are shown in Table 3 and Table 4, respectively. Compared to the classification accuracy (CA) and recall (REC) values returned by the pre-evaluation tests, the performance in the training phase of the MLPBNN machine learning algorithm seemed to improve by half of one percent. In addition, the lowest LOGLOSS errors, as well as the highest values of classification accuracy (CA) and recall (REC), were found for those classes characterizing the static work (class codes ending with “f”, Table 3). In the testing phase, however, the overall classification performance was significantly lower, showing a low ability to generalize over the data.

A similar trend in classification performance was found for the RF machine learning algorithm (Table 4) in the sense that static work was better classified in the training phase (higher CA and REC and lower LOGLOSS). The training phase also showed that the overall values of CA, REC, and LOGLOSS were improved compared to the pre-evaluation test. In the testing phase, however, the classification performance was poorer compared to that of the MLPBNN algorithm.

## 4. Discussion

Detailed data collected over the long term to evaluate body postures and frequency of dynamic and static work are essential in the attempt to understand the mechanisms that govern human biomechanical exposure during work, having several applications in ergonomics and healthcare [56,57,58,59,60]. This study evaluated the possibility of using signals collected by triaxial accelerometers and machine learning techniques to classify the posture of the back and to differentiate dynamic from static work in an experimental setup.

Performance of supervised classification by machine learning algorithms depends largely on the quality of the input signals, labeling quality, and complexity of classification. Quality of the input signals, including here triaxial acceleration, may be quantified by several statistical parameters including the signal-to-noise ratio (SNR) and coefficient of variation (CV) [61]. Improving the SNR of a signal is typically done by signal processing techniques such as filtering, which can be implemented by various types of filters. Following a visual examination of the data in this study, a median filter with a sliding window size of 41 instances has shown a good ability to remove impulses and oscillations from the triaxial signals. On the other hand, the window size of a median filter may largely depend on the signal patterns in either the time or frequency domain, which inherently represent the type of underlying processes being studied. Smaller window sizes would be useful in preventing losses in original data, although several ways of imputing it in the filtered signal have been described by the relevant literature [42]; however, their outcomes in terms of classification performance may differ as a function of the strategy used for filtering. Recent studies, including some that considered acceleration data, have used window sizes of three observations [46,62] with diverging results. For instance, [46] found that simple median filtering by a window size of 3 instances was useful in enhancing the classification accuracy for a process characterized by a rather low variability of acceleration in the time domain. The study of [62], on the other hand, found that filtering-to-the-root by a median filter with a window of 3 instances has brought less utility in the attempt to enhance the classification performance. Since the representation of the signal in the time domain was supported in this study by the finest sampling rate enabled by the datalogger (20 Hz), it was almost impossible to prevent the occurrence of impulse noise and oscillations. Although these types of noise could be subject specific, it is more likely that filtering the data collected inter-individually would provide a common ground for reaching acceptable classification performances for the same architecture of a machine learning algorithm.

In general, labelling the data, particularly with post hoc procedures applied to accelerometer signals, is a challenging task [21]. In this study, the strategy used to provide an external reference for labelling tasks was to record the experiment by a video camera. This strategy was complemented by designing the protocol to be done in slow motion to enable data labelling. Nevertheless, the labelling effort was consistent, as it was based on observing the patterns in the data and cross-checking the events from media files with those from the input data set. As the sampling rate was set to its finest value, there were difficulties to overcome in the labelling effort. Such difficulties arose from the number of classes and the human capability of accurately separating their occurrence in the fine-sampled input dataset. Although the best effort was given to accurately separating the classes in the time domain, there is some uncertainty in relation to the accuracy of labelling that cannot be accounted for. However, further research could be focused on integrating the process of labelling with that of pre-evaluation by machine learning, to check, by an iterative procedure, if improvements could be added to the classification performance.

The complexity of classification depends, among other things, on the patterns found in the signal and the number of classes required by an experiment. Although for the human eye, it could be easy to identify different events in such patterns (which was, in general, the case of this study), supervised machine learning approaches to the problem need to overcome several other limitations, such as the class imbalance, intra-class variability, and inter-class similarity [21]. Accordingly, there is no known best solution in terms of a machine learning algorithm class to be used for a given problem, as there is no known best architecture of an algorithm from the same class to solve that problem. That is the reason why this study selected two classes of machine learning algorithms based on the evidence from previous research (e.g., [23,24]), their statistical properties [44,51,52], and the experience of the authors with them [24,45,46,62], as well as for trying to learn by pre-evaluation from the data by altering the hyperparameters of the used algorithms. However, good results obtained by a pre-evaluation will not absolutely guarantee that a given architecture of the machine learning algorithm will perform similarly in the testing datasets. This was proven by this study for both classes of machine learning algorithms. As shown by the results, at first glance, the RF seemed to highly outperform the MLPBNN machine learning algorithm. However, this did not hold true in the training and testing phases, in which the MLPBNN has preserved a better generalization ability, although it returned lower values in terms of classification accuracy and recall.

The lower performance in the testing phase was less likely to come from the class imbalance issues, which is one of the main challenges in applications of human activity recognition [21]. This is because the data used in both the training and testing phases was relatively balanced. An exception was found for the testing data subset in which the static work accounted for more instances. However, this class was generally characterized by a pattern that did not produce severe misclassifications, at least in the case of the MLPBNN algorithm. In contrast, some differences and uncertainty may have come from the amplitude of movements and variation in movement speed, which, although they were guided, were rather at the control of the subject. To enhance the separability of the data, the datalogger was placed on the subject as far as possible from the thorax–legs joint. This choice was based on the intuition that wider displacements in signals during back movements could be obtained in such a setup. For instance, findings of previous studies indicated a high differentiation between static and dynamic work when the signals were collected at the chest level [31]. Another problem could be that of drifts in the signals caused by location changing of the datalogger on the subject’s body. This last issue can represent a guiding point in finding ways of preserving the invariability of dataloggers’ location, which was difficult to control with the design of this study. Most likely, this effect was recorded in the data collected on the z-axis, which did not follow the same pattern over time and over different types of movement. Therefore, it is possible that variation in movement amplitude and speed, as well as the location drifts, have caused some data misclassification, which ultimately may explain the differences in performance in the three phases: i) pre-evaluation and selection of the models, ii) training, and iii) testing. 

Pre-evaluation of the models has used all the data, allowing the machine learning algorithms to learn on them; therefore, the results of this phase were seen as common, with a high classification performance. Training of the algorithms used approximately 70% of the dataset, capturing most of the variation, including that recorded on the z-axis (see Figure 4 for an example); therefore, the ability to learn was likely to be high. In contrast, only the last three movements of each subprotocol were kept for testing. If systematically or by chance the patterns in these data segments were different, then they would have affected the classification performance in the testing phase. Other misclassification problems could have been produced by the intra-class variability and inter-class similarity [21], which were characteristic of this study (some examples may be seen in Figure 7).

Keeping in mind the limitations described above, the overall results of this study indicate that it is possible to accurately differentiate at least between the dynamic and static work of the back in the time domain by an MLPBNN algorithm applied to a complex experimental setup. For the used dataset, it is likely that a random sampling without replacement of the data in the training and testing subsets would have given a chance for better results, a problem which may be explored further. As there were evident differences in classification performance between the results of the two tested algorithms, it is likely that other classes of machine learning techniques or architectures set for them would produce different results. This is a reason for further exploring the problem of increasing the classification performance. Some tests were done (data not presented in this study) with Bayes classifier (BC) and support vector machines (SVM), as these are well supported by Orange Visual Programming software [43], but at an initial stage, the results were poorer than those of MLPBNN and RF. This does not exclude the possibility that a fine-tuned SVM, for instance, would provide better results. Since the classification performance depends on the information carried by input signals, further work could explore, for an experiment such as that described herein, the possibility of using fewer signals, as well as of using paired combinations of the tree axial signals.

Altogether, median filtering of the triaxial acceleration signals, followed by pre-evaluation of two machine learning algorithms, including hyperparameter tuning, were the main adaptations used in this study to enhance the ability of separating the dynamic and static work, while accounting for the posture of the back. All of these contribute to our knowledge on how acceleration data and machine learning can be used to map the postural profile and separate dynamic and static work, which have important applications in healthcare. MLPBNN performed better, which is an indication that this class of algorithms could be suitable for real-world, unconstrained applications. In particular, static work was more accurately detected, which could be used in real-world applications to infer postural profiles, including by generalization to different subjects characterized by different anthropometrics. This is mainly because the workflow used in this study normalizes the data [43] to balance the variability of observations’ magnitudes in a delimited range before feeding it into the MLPBNN. The computational efficiency, on the other hand, could be one of the limitations of using MLPBNN, at least in the training phase, since the RF generally trains faster. However, for the best architectures found in the pre-evaluation tests, the results on training time (data not shown herein) were comparable. This does not mean that training over larger datasets will preserve this balance in computational efficiency in the training phase. Testing, on the other hand, runs very fast, enabling an efficient offline data classification, assuming that new data would be available. 

Compared to the findings of the previous studies using accelerometers as information collectors, which were typically focused on activity recognition, this study proves that it is possible to use triaxial acceleration signals to correctly classify by machine learning algorithms the posture and dynamic and static work of the back by considering a high number of classes (13 classes tested in this study), an approach that was less common in previous studies. The study also indicates that the classification performance of triaxial signal dilutes as the number of target classes increases (i.e., 2 class vs. 13 class problem), providing a ground for improvements such as selecting other combinations of axial signals, augmentation techniques, or other types of machine learning algorithms for training and testing tasks. Assuming an out-of-lab application of the study concept, it is likely to find similar patterns in the data for similar setups and movements, as the protocol used covered the main degrees of freedom in back movements. Therefore, in jobs characterized by fixed workstations, the approach may be readily available, while in those involving walking, it should be updated by integrating new data.

This study was experimental, so the possibility of training and testing higher-performing machine learning algorithms on real-world collected acceleration data to differentiate between static and dynamic work, and particularly to infer the postures of the back, needs to be pursued further. Depending on the algorithms used, such an attempt would have to rely on large amounts of data, and it would need to include the variability brought by anthropometry and task specificity. This would be a significant challenge in terms of the data labelling effort, for which new solutions are required and which will be provided, most likely, by the advancement in computer vision, unsupervised and deep convolutional learning, or multi-modal sensing.

## 5. Conclusions

Machine learning could be a suitable approach to the problem of postural classification and detection of dynamic and static work of the back in the time domain based on acceleration data. This is supported, in particular, by the results obtained in the pre-evaluation phase of this study, in which the classification accuracy and recall were of 85% to 93%, depending on the type of problem, class of machine learning algorithm used, and its architecture. Although the results obtained in the testing phase indicated a lower generalization performance, as is known to happen, the general performance of a machine learning algorithm in classification problems depends on the amount and quality of data fed to it. Once a sufficient amount of fine-labelled acceleration data becomes available, a lot of potential will be unleashed in detecting and classifying complex body postures in the time domain, including those postures that overlap on both dynamic and static work. In turn, these will enable the capability of building postural profiles for various types of jobs and linking them to the results of epidemiological studies to reach conclusions regarding their effect on human health and to intervene for improvement.

## Figures and Tables

**Table 1:** Description of the coding protocol used in this study.

Feature	Code	Description and Comments
back	B	
straight	s	Includes observations for which the orthostatic posture was maintained, as well as those for which the knees were bent
bent to the left	bl	-
bent to the right	br	-
bent forward	bf	-
bent forward and twisted to the left	btl	-
bent forward and twisted to the right	btr	-
legs	L	-
straight	s	Includes observations for which the orthostatic posture was maintained, as well as those of bending or bending and twisting the back
knees bent	b	Includes observations in which the knees were bent according to the protocol used
presence of movement	P	
true	t	Includes observations in which movements were done according to the protocol (dynamic work)
false	f	Includes observations in which postures were maintained according to the protocol (static work)

**Table 2:** Description of the data subsets used for training and testing.

Dataset	Work Type	Number of Observations	Share in Subset	Share in Data
Training	Dynamic	11,609	49.14	33.50
	Static	12,016	50.86	34.70
	Total	23,625	100.00	68.20
Testing	Dynamic	4,615	41.90	13.30
	Static	6,399	58.10	18.50
	Total	11,014	100.00	31.80

**Table 3:** Classification performance in the training and testing phases to differentiate the postures of the back by the MLPBNN machine learning algorithm with the regularization term set at α = 0.0001.

Phase	Class	Classification Performance Metrics		
		CA	REC	LOGLOSS
Training	Overall	0.855	0.855	0.425
	BbfLsPf	0.993	0.923	0.015
	BbfLsPt	0.969	0.642	0.095
	BblLsPf	0.994	0.986	0.014
	BblLsPt	0.979	0.865	0.052
	BbrLsPf	0.994	0.955	0.016
	BbrLsPt	0.979	0.807	0.059
	BbtlLsPf	0.996	0.974	0.010
	BbtlLsPt	0.974	0.776	0.076
	BbtrLsPf	0.987	0.888	0.026
	BbtrLsPt	0.969	0.716	0.096
	BsLbPf	0.982	0.921	0.038
	BsLbPt	0.951	0.625	0.116
	BsLsPf	0.940	0.978	0.140
Testing	Overall	0.707	0.707	1.142

**Table 4:** Classification performance in the training and testing phases to differentiate the postures of the back by an RF machine learning algorithm with 10,000 trees.

Phase	Class	Classification Performance Metrics		
		CA	REC	LOGLOSS
Training	Overall	0.887	0.887	0.528
	BbfLsPf	0.996	0.949	0.009
	BbfLsPt	0.972	0.722	0.131
	BblLsPf	0.997	0.973	0.008
	BblLsPt	0.983	0.904	0.053
	BbrLsPf	0.997	0.969	0.011
	BbrLsPt	0.984	0.863	0.065
	BbtlLsPf	0.998	0.976	0.011
	BbtlLsPt	0.977	0.821	0.104
	BbtrLsPf	0.994	0.949	0.017
	BbtrLsPt	0.972	0.781	0.110
	BsLbPf	0.989	0.940	0.033
	BsLbPt	0.961	0.732	0.137
	BsLsPf	0.956	0.969	0.151
Testing	Overall	0.615	0.615	2.289

## Data Availability

Data supporting this study may be provided by the authors based on reasonable request.

## Appendix A

**Table A1:** Movement and posture change protocol used in this study.

**Protocol**	**Subprotocol**	**Full Description**
A Full movement at low speed	A1	Maintaining the orthostatic posture for ca. 30 s; Bending the back to the left at full extent; Maintaining this posture for approximately 5 s; Returning to orthostatic posture and maintaining it for ca. 5 s; Repeated 10 times.
	A2	Maintaining the orthostatic posture for ca. 30 s; Bending the back to the right at full extent; Maintaining this posture for approximately 5 s; Returning to orthostatic posture and maintaining it for ca. 5 s; Repeated 10 times.
	A3	Maintaining the orthostatic posture for ca. 30 s; Bending the back forward at full extent; Maintaining this posture for approximately 5 s; Returning to orthostatic posture and maintaining it for ca. 5 s; Repeated 10 times.
	A4	Maintaining the orthostatic posture for ca. 30 s; Flexing the kneels at full extent with the back straight; Maintaining this posture for approximately 5 s; Returning to orthostatic posture and maintaining it for ca. 5 s; Repeated 10 times.
	A5	Maintaining the orthostatic posture for ca. 30 s; Bending and twisting the back to the forward-left at full extent; Maintaining this posture for approximately 5 s; Returning to orthostatic posture and maintaining it for ca. 5 s; Repeated 10 times.
	A6	Maintaining the orthostatic posture for ca. 30 s; Bending and twisting the back to the forward-right at full extent; Maintaining this posture for approximately 5 s; Returning to orthostatic posture and maintaining it for ca. 5 s; Repeated 10 times.
